# Predicting protein and pathway associations for understudied dark kinases using pattern-constrained knowledge graph embedding

**DOI:** 10.7717/peerj.15815

**Published:** 2023-10-18

**Authors:** Mariah V. Salcedo, Nathan Gravel, Abbas Keshavarzi, Liang-Chin Huang, Krzysztof J. Kochut, Natarajan Kannan

**Affiliations:** 1 Department of Biochemistry and Molecular Biology, University of Georgia, Athens, GA, United States of America; 2 Institute of Bioinformatics, University of Georgia, Athens, GA, United States of America; 3 School of Computing, University of Georgia, Athens, GA, United States of America

**Keywords:** Random walk, Illuminating Druggable Genome (IDG), Pathway prediction, Data integration, Ontologies, Link prediction, Classification, Evolution, Signaling networks, Drug discovery

## Abstract

The 534 protein kinases encoded in the human genome constitute a large druggable class of proteins that include both well-studied and understudied “dark” members. Accurate prediction of dark kinase functions is a major bioinformatics challenge. Here, we employ a graph mining approach that uses the evolutionary and functional context encoded in knowledge graphs (KGs) to predict protein and pathway associations for understudied kinases. We propose a new scalable graph embedding approach, RegPattern2Vec, which employs regular pattern constrained random walks to sample diverse aspects of node context within a KG flexibly. RegPattern2Vec learns functional representations of kinases, interacting partners, post-translational modifications, pathways, cellular localization, and chemical interactions from a kinase-centric KG that integrates and conceptualizes data from curated heterogeneous data resources. By contextualizing information relevant to prediction, RegPattern2Vec improves accuracy and efficiency in comparison to other random walk-based graph embedding approaches. We show that the predictions produced by our model overlap with pathway enrichment data produced using experimentally validated Protein-Protein Interaction (PPI) data from both publicly available databases and experimental datasets not used in training. Our model also has the advantage of using the collected random walks as biological context to interpret the predicted protein-pathway associations. We provide high-confidence pathway predictions for 34 dark kinases and present three case studies in which analysis of meta-paths associated with the prediction enables biological interpretation. Overall, RegPattern2Vec efficiently samples multiple node types for link prediction on biological knowledge graphs and the predicted associations between understudied kinases, pseudokinases, and known pathways serve as a conceptual starting point for hypothesis generation and testing.

## Introduction

Protein kinases play a fundamental role in cell signaling by phosphorylating peptides and proteins on serine, threonine, and tyrosine residues. The 534 protein kinases encoded in the human genome regulate protein activity in diverse pathways including those involved in DNA repair, cell cycle control, and metabolism (Hunter, 2009; Johnson et al., 2023; Lahiry et al., 2010; Ochoa et al., 2020). Because dysregulation of protein kinase signaling is causally associated with the pathogenesis of many diseases (Ardito et al., 2017; Oprea et al., 2018), much effort has gone into the characterization of their physiological functions (Ayala-Aguilera et al., 2022; Xie et al., 2021) and the development of selective protein kinase inhibitors (Ardito et al., 2017; Attwood et al., 2021; Xie et al., 2021). Despite these efforts, nearly 164 out of the 534 known kinases remain relatively understudied and are referred to as “dark” kinases by the NIH Illuminating the Druggable Genome consortium (IDG) (Berginski et al., 2021; Gillespie et al., 2022; Kelleher et al., 2023). Characterizing the functions of these dark kinases is crucial because they work in conjunction with other well-studied kinases in signaling pathways and are also frequently mutated, or abnormally expressed, in human diseases such as cancers (Berginski et al., 2021; Brognard & Hunter, 2011; Collins et al., 2018; Ravanmehr et al., 2021; Soleymani et al., 2022). While considerable progress has been made in illuminating the functions of several dark kinases, placing these kinases in a pathway or a cell signaling network context remains a major bioinformatics challenge.

The compendium of genomic, proteomic and interactome data now available through the efforts of the IDG consortium and numerous investigator-initiated efforts allows for the possibility of inferring the functions and pathways for dark kinases through integrative mining of the known patterns and relationships in existing data. In particular, the development of network-based approaches, such as knowledge graphs (KGs), that relate and link diverse types of protein kinase data in the forms of networks (composed of nodes and edges) enables the prediction of dark kinase functions or pathways through network context (Soleymani et al., 2022). Indeed, KG mining approaches and machine learning (ML) methods applied to KGs have been successfully employed in the identification of kinase substrates (Gavali et al., 2022; Nováček et al., 2020), prioritization of understudied kinases (Huang et al., 2018), and identification of disease associations (Bachman, Gyori & Sorger, 2022; Gyori et al., 2017; Moret et al., 2021; Soleymani et al., 2022).

The working premise of these network-based approaches is that proteins (kinases in this case) that share similar network neighbors (such as interacting proteins, downstream substrates, cellular localization, or molecular functions) with well-studied kinases are likely to be involved in common pathways. Several predictive ML methods utilize this premise, however many of these methods are designed for use with homogenous networks (Grover & Leskovec, 2016; Perozzi, Al-Rfou & Skiena, 2014). Homogenous networks have just a single node and edge type. Whereas heterogenous networks, like KGs, are defined by multiple node and relationship types such as *Drug–targets–Protein* and *Protein–isAssociatedWith–DiseaseState,* where *Drug* and *Protein* are node types and *targets* and *isAssociatedWith* are edge types (or relationships). Because heterogenous networks offer a broader range of knowledge representation over homogenous networks, they are suitable for biological representations that require data integration and conceptualization from multiple data sources. However, models that can fully leverage heterogenous graphs often suffer from scalability issues, limiting their predictive power and making the construction of the KG difficult (Alshahrani, Thafar & Essack, 2021; Bonner et al., 2022; Gavali et al., 2022). Due to these limitations, KGs have not been previously employed to predict pathway associations for dark kinases because they are sparsely connected in KGs (due to low information content), making it difficult to predict functional associations through local context alone.

Embedding-based approaches have been proposed to overcome the limitations of sparse data within KGs, as they have the capability to represent the graph in dense and low dimensional feature space (Dai et al., 2020). However, many seminal methods developed for graph embedding are not optimized for heterogenous graphs as is the case with many message-passing GNN-based models (Hamilton, Ying & Leskovec, 2017; Pei et al., 2020; Tang, Li & Yu, 2019; Velickovic et al., 2017; Zhang et al., 2018; Zhang et al., 2019). Other methods, such as proximity-preserving methods, have had success in large-scale knowledge graph embedding, however using these methods to fully capture complex relations is still an active area of research as many of these models lack full expressivity (accounting for both local and global dependencies) (Bordes et al., 2013; Gao et al., 2020; Ge et al., 2022; Huang et al., 2021; Sadeghian et al., 2021; Sun et al., 2019; Wang et al., 2021; Yang & Liu, 2021; Zhou, Yi & Jia, 2021). However, a recent advancement, the spatio-translational model BoxE, is fully expressive, but due to the model’s use of the “rule injection” method, yields high false positivity rates with sparse graphs (Abboud et al., 2020).

Relation-preserving methods attempt to capture complex relationships by matching semantic similarities of entities and relations and learn “context” from nodes within the KG. Many base-line relation-preserving methods are still widely used for link prediction tasks with biological data (Chen et al., 2020; Gan et al. 2023; Ha & Park, 2022; Li et al., 2020; Long & Luo, 2020; Peng, Guan & Shang, 2019; Samizadeh & Minaei-Bidgoli, 2020; Wang et al., 2021; Wong et al., 2020). This deep learning approach is based on a family of models from Natural Language Processing (NLP) called word2vec and can be combined with constrained random walks to sample the graph and allow for use with heterogenous networks (Dong, Chawla & Swami, 2017). By sampling subsections of the KG, random walk-based methods can scale with larger KGs that would otherwise be too computationally expensive to use in full. While many techniques are used for data reduction in KGs (Wang et al., 2017), constrained random walks have distinct advantages. Using a constrained “random walk” method on the KG, the graph is stochastically “sampled” such that multiple data types present within the network can be appropriately leveraged for predictions. The sampled section of the graph can then be converted into an embedding of the KG such that the biological context relevant for the prediction is accurately captured enabling nontrivial associations between dark kinase and associated pathways (Dong, Chawla & Swami, 2017; Nickel et al., 2015).

One of the most well-known relation-preserving methods that utilizes random walks is metpath2vec. metapath2vec preserves the relationships in the graph during the sampling process by utilizing schemas (ordered sets of specific node or edge types). By specifying what node/edge types should be sampled, integration of different data types can be fully leveraged for predictions, thus accurately capturing the structure of heterogenous graphs. However, creating such meta-paths for complex heterogeneous KGs, often with no well-defined schema, is challenging and time-consuming with the performance of the model being highly dependent on the chosen meta-path (series of nodes).

Several previous attempts to automatically generate meta-paths have been reported in the literature (Meng et al., 2015; Wan et al., 2020; Yang et al., 2018) but they all rely on fixed-length meta-paths, which prevents accurate representation of latent and hierarchical structure encoded in large KGs. Other works have expanded upon the random walk framework proposed in metapath2vec, including W-MetaPath2Vec (Pham & Do, 2019), HIN2Vec (Fu, Lee & Lei, 2017), and RW-k (Anil et al., 2019). Additional works have combined the use of meta-path-based sampling with graph neural networks (GNNs) to make the aggregated message passing method more appropriate with heterogenous networks, as is the case with MAGNN (Fu et al., 2020). Finally, attempts to exploit semantics explicit in structural relations found within the graph have been leveraged for graph sampling, as is the case with Relation Structure-Aware Heterogenous Information Network Embedding (RHINE) (Shi et al., 2020), which defines relations that link nodes according to their similar properties or ability to bridge compatible nodes. We explore several of the methods listed above (MAGNN, RHINE, BoxE) for use with our graph (See Results ‘Benchmarking RegPattern2Vec’s predictive performance with metapath2vec and other graph embedding approaches’). However due to the focus of our question relating to the functionality of dark kinases there is inherent data imbalance within the graph making these methods not appropriate for use.

To address this, we employ RegPattern2Vec (Keshavarzi, Kannan & Kochut, 2021), a novel approach for learning on semantic data to predict new pathway associations for dark kinases. RegPattern2Vec directly addresses the limitations of previous models and provides more accurate predictions by precisely capturing relevant node/edge context in areas of graph sparsity using regular patterns and hyperparameters (See Methods & Materials ‘Regular pattern selection and its usage in random walks’–Biased random walk constrained by a regular pattern). Unlike metapath2vec, RegPattern2Vec can make node-association predictions without specifying the entire schema. This allows us to meaningfully sample the KG without establishing multiple meta-paths (Keshavarzi, Kannan & Kochut, 2021). Here we show the application of RegPattern2Vec for link prediction on a large heterogenous KG.

Overall, we successfully employed RegPattern2Vec to capture the node/edge context more accurately within our KG. After sampling, the ML technique (link prediction) is used to predict new associations between dark kinases and characterized pathways. We place 34 dark kinases for which we have consistent, high-confidence predictions produced through replicate runs (with differing hyperparameters) in a pathway context and based on an analysis of the meta-paths navigated for three selected dark kinases, we provide biological interpretations of the predicted pathway associations. The RegPattern2Vec pipeline is available at the GitHub repository (https://github.com/gravelCompBio/RegPattern2Vec).

## Materials & Methods

### Knowledge graph architecture

KGs are very similar to Heterogeneous Information Networks. An Information Network is a directed graph $G= \left( V,E \right) $, composed of vertices (also called nodes) and edges, with an associated node type mapping function *ϕ*:*V* → *A* and an edge type mapping function *ψ*:*E* → *R* (Shi et al., 2016). Each node *v* ∈ *V* belongs to one particular node type in the node type set $A:\phi \left( v \right) \in A$, and each edge *e* ∈ *E* belongs to a particular edge type in the edge type set $R:\psi \left( e \right) \in R$. An Information Network is called a Heterogeneous Information Network if the sets A and R both contain more than one element, that is, there are multiple labels (types) for graph nodes and multiple labels (types) for edges. If sets A and R are singletons, the Information Network is called a Homogeneous Information Network (all nodes in the network are of the same type, and all edges are of the same type). While Heterogeneous Information Networks require that if two edges belong to the same edge type, the two edges share the same starting node type and ending node type, KGs do not. That is, the same edge (relation) type can be applied to different starting node types and different ending node types. This is also the case in the Resource Description Framework (RDF) (Cyganiak et al., 2014; W3C, 2014), a notation often used to represent KGs. RDF is based on the notion of triples of the form *Subject*-*predicate*-*Object* (Cyganiak et al., 2014) representing edges connecting nodes in the graph.

**Figure 1:**
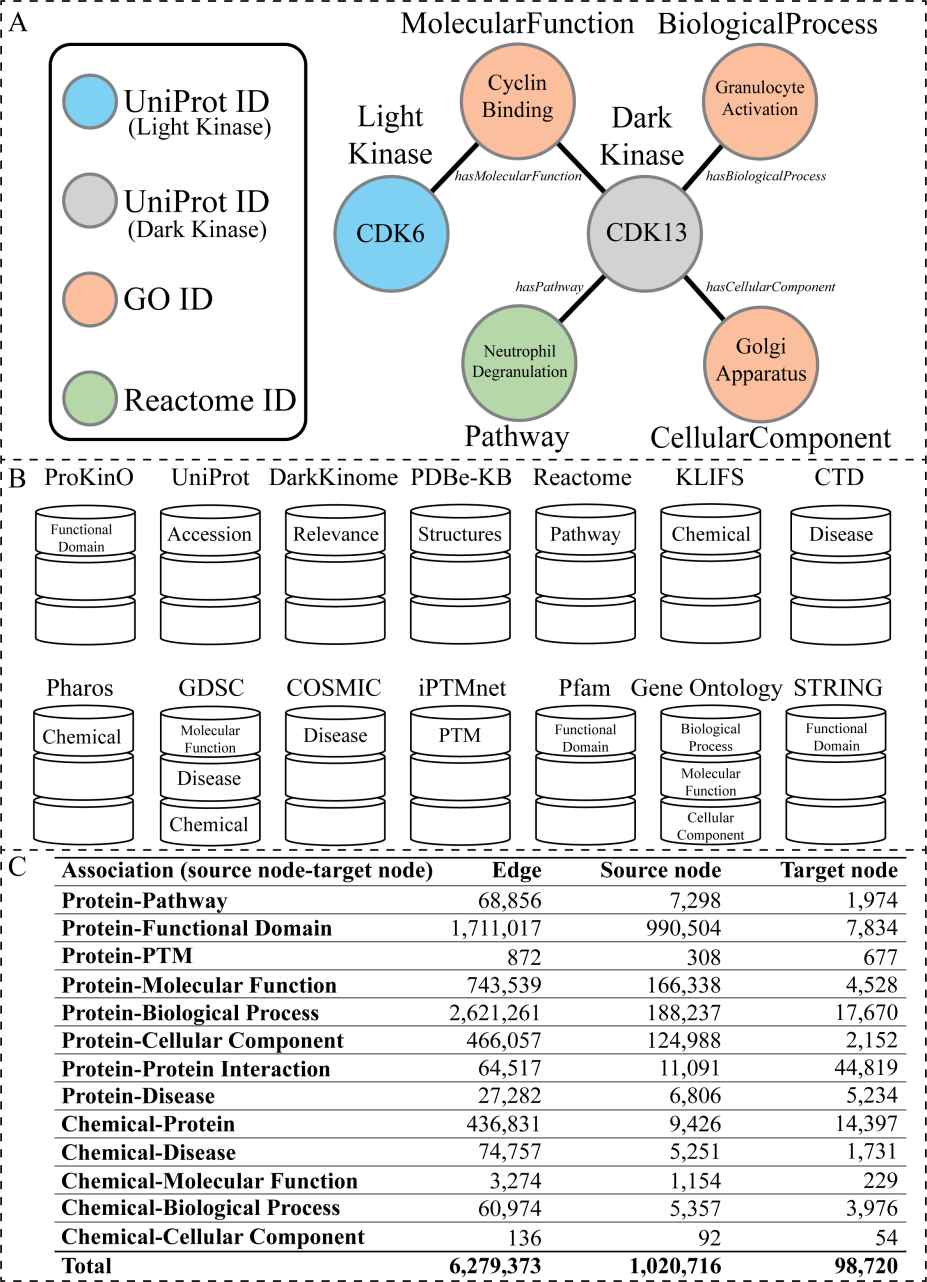
Overview of the KG. (A) Visual representation of the node types present within the KG generated from the listed databases above. The circles represent nodes (entities), and the lines represent edges (relationships). The circles are colored according to node type. The labels inside the circle are specific examples of data found in our KG related to the dark kinase CDK13. The labels outside of the circles correspond to the node type in bold text and the edge type in italics. (B) A graphical representation of all databases utilized in the construction of the KG with the node type label listed inside the representation of each corresponding database. (C) Abundance of associations with corresponding edge and node counts that exist in the KG.

For example, in Fig. 1A, for a KG representing information about protein kinases, a node (entity) representing a dark protein kinase *CDK* 13 (cyclin dependent kinase 13) can be connected by an edge (relationship) labeled *hasPathway* to a node representing a pathway *NeutrophilDegranulation*, which represents the knowledge that *CDK* 13 participates in the pathway *NeutrophilDegranulation*. In such a KG, *CDK* 13 may be connected to other nodes using different labels (*hasPathway*), such as *CDK13–has*Molelcular*Function—CyclinBinding*, *CDK13–hasBiologicalProcess—GranulocyteActivation* and *CDK13–hasCellularComponent—GolgiApparatus*. Other protein kinases can additionally be included in the neighborhood context through shared nodes, such as the shared molecular function of *CyclinBinding* between dark kinase *CDK13* and light kinase *CDK6* (cyclin-dependent kinase 6). Here, edges have multiple labels, and destination nodes are of different types, which indicates that it is a heterogeneous KG.

Given a Heterogeneous Information Network $G= \left( V,E \right) $ (as defined above), the network’s schema is a directed graph, $S= \left( A,R \right) $, based on G’s node type mapping *ϕ*:*V* → *A* and its edge (relation) type mapping *ψ*:*E* → *R*.*S* is a directed graph defined over node types *A*, with edges as relations from *R*. Similarly, the schema of a KG represented in RDF is represented in RDFS (RDF Schema (W3C, 2014)). For example, given the *CDK13* examples above, the schema would contain the edges *Protein —participatesIn—Pathway*, *Protein–hasBiologicalProcess—BiologicalProcess,* and *Protein—isLocatedIn—CellularLocation*. The schema, sometimes referred to as a meta-knowledge graph, or meta-graph, specifies constraints on using the edge labels to certain types of starting and ending nodes (subjects and objects in RDF). Also, given a KG schema, we can create a KG conforming to the schema containing many individuals (nodes).

### Data sources and curation of datasets for knowledge graph generation

**Figure 2:**
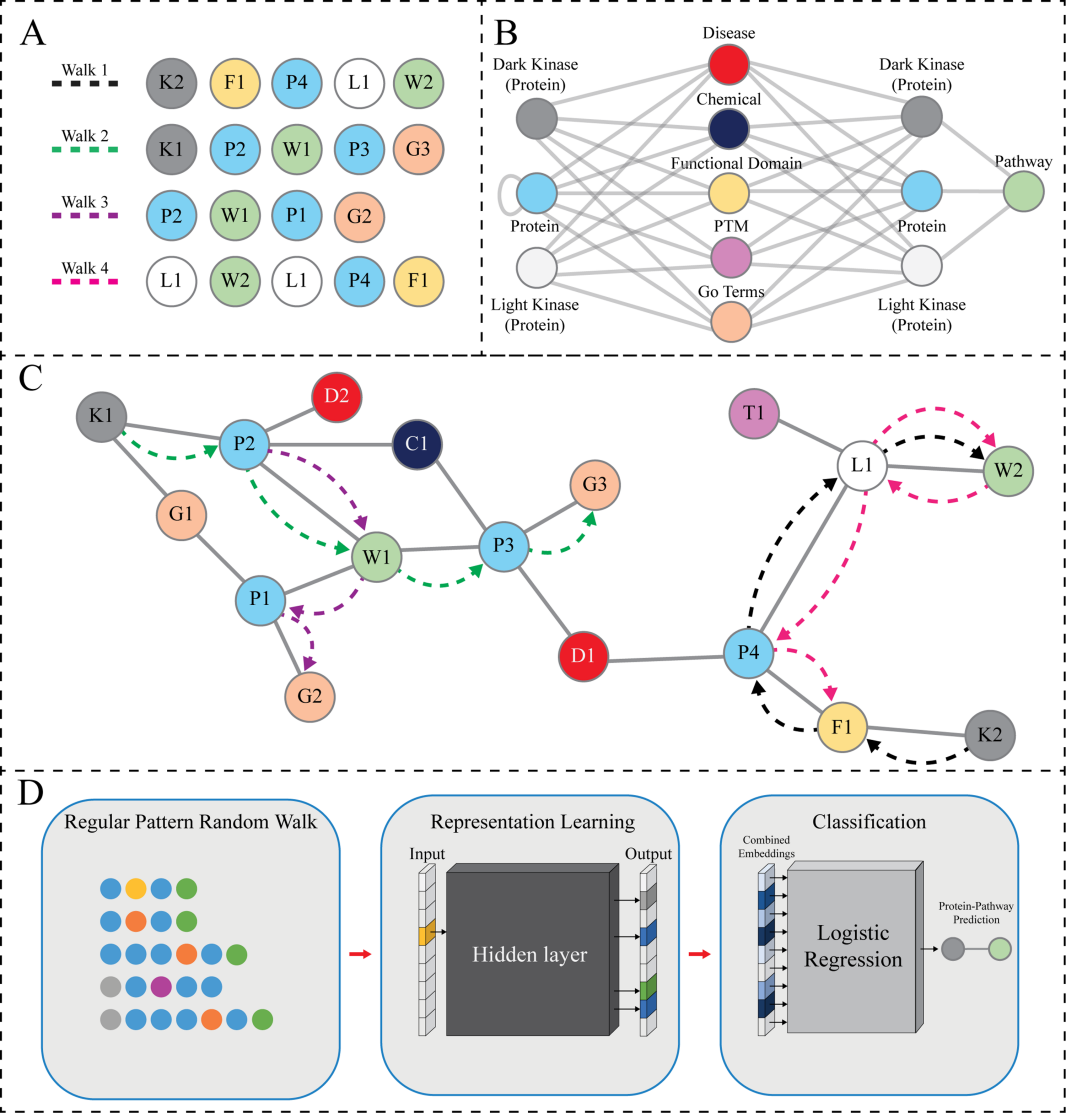
RegPattern2Vec Overview. (A) The hypothetical sequence of nodes produced by random walks guided by the regular pattern, shown in (B) (see legend for panel B for more details) and with the limitation of collecting five nodes (hyperparameter Length of Walk). This can result in several walks shown in (A). Walk1 shows a full walk that ends on a pathway node, Walk2 shows a full walk that ends on a node other than pathway, Walk3 shows a walk that ends early, and finally Walk4 shows a reverse walk. (B) The regular pattern designed for random walk sampling (schema). This pattern is defined as *Protein* [^∧^*Pathway* ] + *Protein Pathway*. Each random walk instance is constrained based on this pattern. All meta-paths must follow the schema in which they start with either a DarkKinase, LightKinase or Protein node and must collect a pathway node during the random walk. Once a pathway node is collected, if there are no other appropriate nodes according to the limitations put into place by both the schema and hyperparameters then reverse walks are allowed in which the pathway node will be collected again, and the walk resumes in a random direction until hyperparameters are satisfied. This feature is added to capture local relevant nodes in areas of data sparsity. (C) A hypothetical subgraph of the KG. The dashed lines show the node sequences highlighted in (A). Nodes are labeled and colored according to node types as shown in panel B with numbering corresponding to the order in which it is sampled (IE: P3 is the third Protein node sampled). This figure serves as a graphical depiction of how various data types exist within a neighborhood for sampling. (D) The meta-paths produced through our modified random walk approach are turned into embeddings for representation learning. Once the node context has been vectorized we then use logistic regression to perform link prediction as a binary classification task.

To construct our human kinase-centric KG, we gathered data from multiple publicly available curated resources (Fig. 1B). Nodes and edges within the graph were further integrated under more general node and relationship labels (also referred to as node types and edge types respectively) such as *Disease* (Figs. 1B and 2B). We further filtered the information included within our KG to avoid redundancy (described below). Fig. 1C shows basic statistics of different edges and their source and target nodes. The final KG contained 1,064,097 nodes of 11 types and 6, 279, 374 associations of 13 types.

The node types *Protein* and *FunctionalDomain* were populated with information on human kinase classification, functional domains, structure, and Protein–Protein Interaction (PPI). Information on these data types was extracted from the following databases: ProKinO (McSkimming et al., 2015), Dark Kinase Knowledgebase (darkkinome.org) (Berginski et al., 2021), UniProt (UniProt, 2021), PDBe-KB (Velankar et al., 2010), Pfam v33.1 (Mistry et al., 2021), and STRING v11 (Szklarczyk et al., 2019). Of note, included within our graph are numerous ortholog (non-human) *Protein* nodes collected from PPI databases. *Protein* Post-Translational Modifications (PTMs) associations were also included within our KG under node type *PTM* and were retrieved from iPTMnet v5 (Ross et al., 2017). The *Chemical* and *Disease* node types were populated from the Comparative Toxicogenomics Database (Davis et al., 2021) (protein-chemical, protein-disease, chemical-disease, and chemical-GO term associations) as well as cancer mutation data from the Catalogue of Somatic Mutations in Cancer (COSMIC) (Tate et al., 2019). The Genomics of Drug Sensitivity in Cancer (GDSC) (Yang et al., 2013) database was used for drug activity data. The *Chemical* node type additionally includes information on kinase-associated and ligand interaction motifs and ligand activity retrieved from Kinase-Ligand Interaction Fingerprints and Structures (KLIFS) (Kanev et al., 2021) and Pharos (Sheils et al., 2021), respectively. Gene ontology terms and pathway information regarding human kinases were retrieved from the Gene Ontology v2019_11 (Gene Ontology, 2021) (*GOTerm* node type) and Reactome v76 (Jassal et al., 2020) (*Pathway* node type), respectively.

### Further filtering of data sources to reduce redundancy

The retrieved datasets were further curated prior to KG generation and population. All protein-pathway associations obtained from Reactome were split into manually curated associations (evidence = TAS) and predicted associations (evidence = IEA), only manually curated associations were included in our KG. Additionally, due to the hierarchical nature of the data taken from Reactome (i.e., Pathways are often organized into parent–child relationships), we eliminated high level pathways as they were deemed too general for our experiments. For example, we filtered out the pathways beneath “Disease” (R-HSA-1643685) but not beneath “Infectious disease” (R-HSA-5663205). This was done not only to reduce redundancy in the data but also to ensure the vector embeddings created from our KG for link prediction were not overfitted to highly connected nodes (often referred to as *hubs*). Similar filtering was done with Protein-GO term associations retrieved from Gene Ontology-GO terms with more than 5000 associations were removed. Additionally, Protein-GO terms were assigned separate node and edge types in the KG according to the *MolecularFunction*, *CellularComponent*, and *BiologicalProcess* terms.

Additional filtering to increase confidence in the data within our KG was performed using various strategies specific to the data source. Only PPIs (STRING) directly involving a kinase and with an experimental data score of more than 700 were included in our KG, and only PTMs (iPTMnet) with a confidence score of more than 1.0 were included in the graph. Additionally, Pfam entries with the protein kinase domains (“Pkinase” and “Pkinase_Tyr”) were removed.

### Creation of curated kinase knowledge graph for kinase-pathway link prediction

The KG used in our link prediction experiments described here contains several node types such as *Protein*, GO Terms (*BiologicalProcess*, *MolecularFunction*, and *CellularComponent), Disease*, and other types. The nodes collected during a hypothetical random walk are shown in Fig. 2A. The hypothetical random walk is constrained by a schema organization that is shown in Fig. 2B the same schema is applied throughout the rest of the paper which indicates [*Protein,* any node not *Protein* and not *Pathway, Protein, Pathway]* (Jassal et al., 2020). In addition, the *Protein* type includes the understudied kinases (referred to as *DarkKinases*), well-studied kinases (referred to as *LightKinases*), and other proteins. This distinction is essential, as it gives us the ability to make predictions for a subset of proteins. The edge types (edges in the schema) are not labeled here, as we do not consider them in our method described here. Instead, we only rely on the types of source and target nodes. The loop edge returning to the *Protein* type indicates the *Protein–Protein* Interaction relation, which is included in our knowledge graph. Finally, Fig. 2C depicts the hypothetical subgraph in which the random walks are performed relating back to the nodes displayed in Fig. 2A.

### Link prediction workflow

In the experiments and results presented in this paper, we used RegPattern2Vec. It is used as the first step in our link prediction process to produce vector representations for the nodes in the graph and formulate the link prediction as a classification problem. A ML model was trained using the combined vectors of existing pairs of nodes connected by an edge (link) of interest. The outline of the method is illustrated in Fig. 2D. We discuss each step of our link prediction method in the subsequent sections.

### Regular pattern selection and its usage in random walks

Given a Heterogenous Information Network (as defined above), we have used regular patterns of the form ***H***[^∧^ ***T***]+ ***H*** ***T*** for link prediction tasks, *i.e.,* edges (links) between nodes of type *H* and *T*. In the pattern, *H* is a source node type and *T* is a target node type of the link of interest, respectively. Additionally, [^∧^T] denotes any node type different from *T* (in regular expressions terminology it is known as the complement operator), and [^∧^T]*+* denotes a repetition (sequence) of one or more node types that are different from *T*. The overall pattern requires that any random walk must match the specified sequence of node types, in that a walk must begin with a node of type *H* then it must be followed by one or more nodes of type different than *T*, then a node of type *H*, which must then be immediately followed by a node of type *T*.

Simply put, the regular pattern is defined as a schema subgraph that should include only the relevant node types for a specific problem. An example regular pattern subgraph is shown in Fig. 2C. Unlike in the metapath2vec method (Dong, Chawla & Swami, 2017), the schema graph pattern represents multiple paths (walks) that can be used to predict missing links. Here, we aim to predict protein-pathway links and the random walks of interest are constrained by a regular pattern connecting node types and edge types most relevant to the prediction links. Here, when generating protein-pathway predictions, we have defined the regular pattern as *Protein [*^∧^*Pathway]* + *Protein Pathway,* and each random walk instance must match it. Each walk starts from a Protein node. Selecting the next nodes on a walk is based on the existing graph nodes and matching the neighbor’s type in the regular pattern. When a random walk reaches a Pathway node, it follows the pattern in reverse order until a certain number of steps (nodes) in the walk are reached, or when it reaches a termination node, *i.e.,* when there is no neighbor to match the pattern. We designed this specific aspect of the model due to the challenge of data sparsity for dark kinases. By allowing the reverse pattern to be followed we allow for additional relevant neighborhood nodes to be collected- when walking backwards once the pathway node is reached, the walk travels in a random direction until the hyperparameters are satisfied, capturing potentially relevant neighbors for our prediction task.

### Biased random walk constrained by a regular pattern

As this work considered undirected graphs, enumerating a given graph’s path is computationally infeasible. The solution is to sample a subset of paths from all possible paths from a random distribution. The random walk constrained by a regular pattern is selected to generate walks of arbitrary length controlled by the “*walk length”* hyperparameter. Having the undirected heterogeneous network and a selected regular pattern, the random walk can be started from each instance of a starting node type in the pattern. As we want all the nodes to appear in our walks, iterating over them would be desirable. It is obvious that if we repeat the walk from each node (not all KG nodes may begin a walk, as constrained by the regular pattern), we will discover more walks as the node might link to multiple nodes of the same type. We call this hyperparameter the “*number of walks”* (referred to as NW in Results ‘Benchmarking RegPattern2Vec’s predictive performance with metapath2vec and other Graph Embedding Approaches’–Investigating the impact of hyperparameters on regpattern2vec’s predictive variability among replicate runs’). We will discuss how to choose the hyperparameter and the analysis of their impact in the Results ‘Benchmarking RegPattern2Vec’s predictive performance with metapath2vec and other Graph Embedding Approaches’–Investigating the impact of hyperparameters on regpattern2vec’s predictive variability among replicate runs’. The next step for each node is to select a node from the adjacent nodes based on the established regular pattern. This might result in multiple choices, and this is where randomization comes to play. RegPattern2Vec can utilize random distribution created by a user-defined function to generate the same probability for all the nodes or use an arbitrary distribution. To implement the random walk collection process, a regular pattern is converted to a Deterministic Finite Automaton (DFA), denoted by M. Each possible walk step is mapped to the DFA. The DFA M is used to check if transitions are allowed (an edge between two nodes); hence, a disallowed change gets a zero probability and is not used in the random walks.

On the other hand, in scale-free networks where the degree distribution follows the power law, some nodes may have a high degree of incoming/outgoing edges (known as *hubs*). Because such high degree nodes can dominate random walks and, consequently, representation learning, one popular way to address this issue is to bias the walks by the inverse of degrees of nodes (Grover & Leskovec, 2016), where the probability of choosing a node *υ*^*i*+1^from *υ*^*i*^ is calculated by normalizing the inverse of degrees of all neighbors of *υ*^*i*^. Although this approach lowers the probability of selecting high-degree nodes, it biases the random walk toward low-degree nodes, thereby capturing pertinent information related to low-degree “dark kinase” nodes. We used the formula below for node selection:

\begin{eqnarray*}P \left( {v}^{i+1}{|}{v}^{i},M \right) = \left\{ \begin{array}{@{}l@{}} \displaystyle \mathrm{g} \left( {r}^{i} \right) \frac{ \frac{1}{ \left\vert {N}_{{v}^{i+1}} \right\vert } }{\sum _{v\in {N}_{{v}^{i}}} \frac{1}{ \left\vert {N}_{v} \right\vert } }   \left( {v}^{i},{r}^{i},{v}^{i+1} \right) \text{is an edge in the graph and the}\\ \displaystyle  \text{transition from v to v is allowed in M} \\ \displaystyle 0  \left( {v}^{i},{r}^{i},{v}^{i+1} \right) \text{is an edge, but the transition is undefined} \\ \displaystyle 0  \left( {v}^{i},{r}^{i},{v}^{i+1} \right) \text{does not exist in the graph}  \end{array} \right. \end{eqnarray*}

In the formula, *υ*^*i*^ denotes the current node and the candidate node for next step is *υ*^*i*+1^ ∈ *N*_*υ*^*i*^_.∣*N*_*υ*_∣ denotes the degree of node *υ*,  and *N*_*υ*^*i*^_ is the set of all the neighbors of node ${\upsilon }^{i}.g \left( {r}^{i} \right) $ is the proportion of the edges of type r ^i^ among all edges of node *υ*^*i*^. Therefore, we randomly choose one edge (relation) type and then use the probability distribution by the inverse of node degrees to select the next node in the walk, but only among the nodes connected by the edge type chosen.

### Vectorization of nodes and deep learning of semantic relationships between proteins and pathways

RegPattern2Vec uses a modified skip-gram model presented in (Dong, Chawla & Swami, 2017) to generate vector representations for the nodes of the KG. The random walks generate sequences of nodes, which resemble natural language sentences. The ML model simultaneously captures the local structure of the graph and the types of the nodes and encodes them as vector representations.

### Link prediction as a classification problem

For each pair of protein-pathway associations, we combined their vector embeddings utilizing a widely used Hadamard product (Grover & Leskovec, 2016; Lin et al., 2015; Minervini et al., 2015). The resulting vector is used as features to train a Logistic Regression model. Training the model additionally requires generating negative examples for protein-pathway association pairs which are difficult as resources with true biological negatives, such as Negatome (Blohm et al., 2014), do not exist for our *Pathway* data type. To generate negative examples, we must first work under the closed-world assumption. This assumes that all information needed is provided, so for a statement to be true it must be explicitly stated to be so. Thus, a lack of a statement denotes that it is false (this contrasts with the open-world assumption in which statements can be true without them being explicitly known to be so). For our relation of interest (predicted link), we randomly select a head and tail node not connected by an edge in the graph. The embeddings of such nodes are then combined to produce negative examples. This process is repeated until the number of negative examples matches the positive examples.

Our logistic regression method utilizes the one-versus-rest strategy, in which a multi-class classification is split into one binary classification problem per class, meaning a classifier will be trained for each task, and therefore all the other data points in the split data are used as negative examples with the methods discussed above (under the closed-world assumption). Thus, the number of negative examples is dependent on the number of positive examples for each classification task. Although our dataset is large, this strategy is employed due to the inherent data imbalance between well-studied and dark kinases.

## Results

In this work, we propose a new guided random walk approach for KGs, known as RegPattern2Vec, used to predict pathway associations for dark kinases. To generate protein-pathway predictions, we first constructed a protein kinase KG by integrating curated data from various resources populating the graph with nodes on molecular function, disease association, protein–protein associations and more (see Materials & Methods ‘Data sources and curation of datasets for knowledge graph generation’). The resulting KG consisted of 1,064,097 nodes (entities) and 6, 279, 373 edges (relationships) (Table S1, Fig. 1C). RegPattern2Vec was then used to sample the large KG into sequences of nodes. Regular patterns from RegPattern2Vec guided sampling. Using such patterns, we generate several walk paths (or a list of sequential nodes in the network) for representation learning. This allows the sampling to capture different sub-structures in the graph (covering different meta-paths) without explicitly designing walks for them.

After sampling, the model learned vector embeddings for each node in the collected paths, utilizing a Natural Language Processing (NLP) model similar to metapath2vec (Dong, Chawla & Swami, 2017). Link predictions utilizing the embeddings were then formulated as binary classification tasks and predictions were made for protein-pathway associations using a logistic regression model. For more details, refer to the Materials & Methods ‘Link prediction as a classification problem’.

### Capturing semantic relationships between proteins and pathways in deep learning

**Figure 3:**
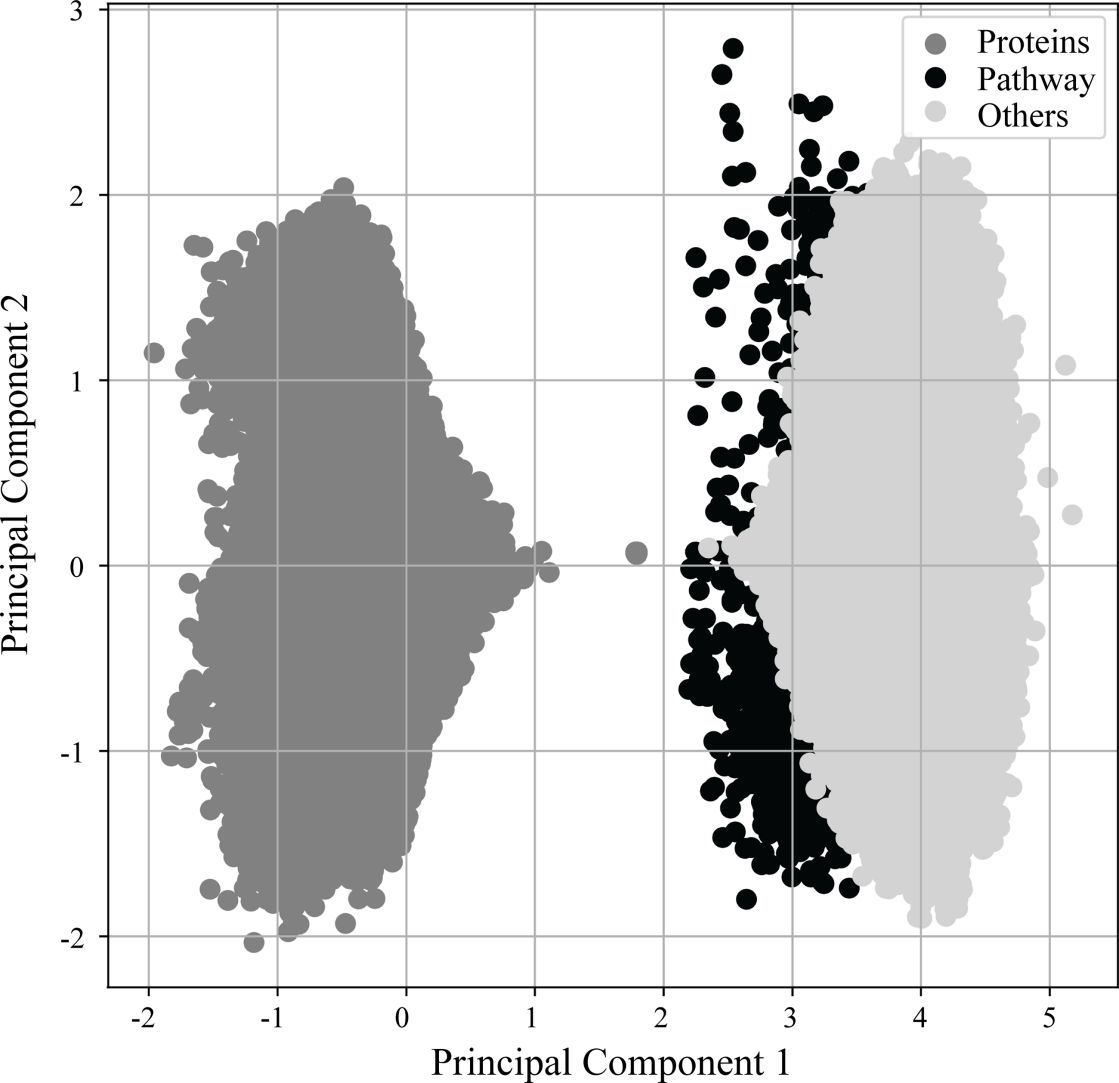
PCA of vector embeddings. Principle component analysis of the vector embedding produced for all nodes generated by ML model. Given that the defined schema of RegPattern2Vec is defined as *Protein* [^∧^*Pathway* ] + *Protein Pathway* when clustering the vector embeddings, we considered all nodes to be of type: *Protein*, anything that is not a *Pathway* or *Protein* (*Others*), and *Pathways*.

As previously mentioned in more detail, (see Materials & Methods ‘Vectorization of nodes and deep learning of semantic relationships between proteins and pathways’) RegPattern2Vec uses a modified skip-gram model presented in (Dong, Chawla & Swami, 2017) to generate vector representations for the nodes of the KG. Figure 3 shows the learned vector representation of nodes in the vector space using principal component analysis (PCA), a standard dimensionality reduction technique. For the protein-pathway predictions, we just consider the nodes to be of three types: “*Protein”*, “*Pathway”,* and “*Others*” when learning representation for the nodes. The separation of nodes in PCA shows our vector representation captures the node types and their network context. Several key differences exist between protein nodes and other nodes in our graph that may explain why they cluster separately from all other nodes. Protein nodes (including those annotated as dark/light kinases) are the default starting node in the schema, and many edge types describe proteins and connect them to other nodes in our graph (for example, a node describing a functional domain will be connected to the protein node it is describing and we additionally illustrate protein–protein interactions in our graph). This may allow protein nodes to occupy a unique space within the embedding reflected in the PCA.

### Benchmarking RegPattern2Vec’s predictive performance with metapath2vec and other graph embedding approaches

**Figure 4:**
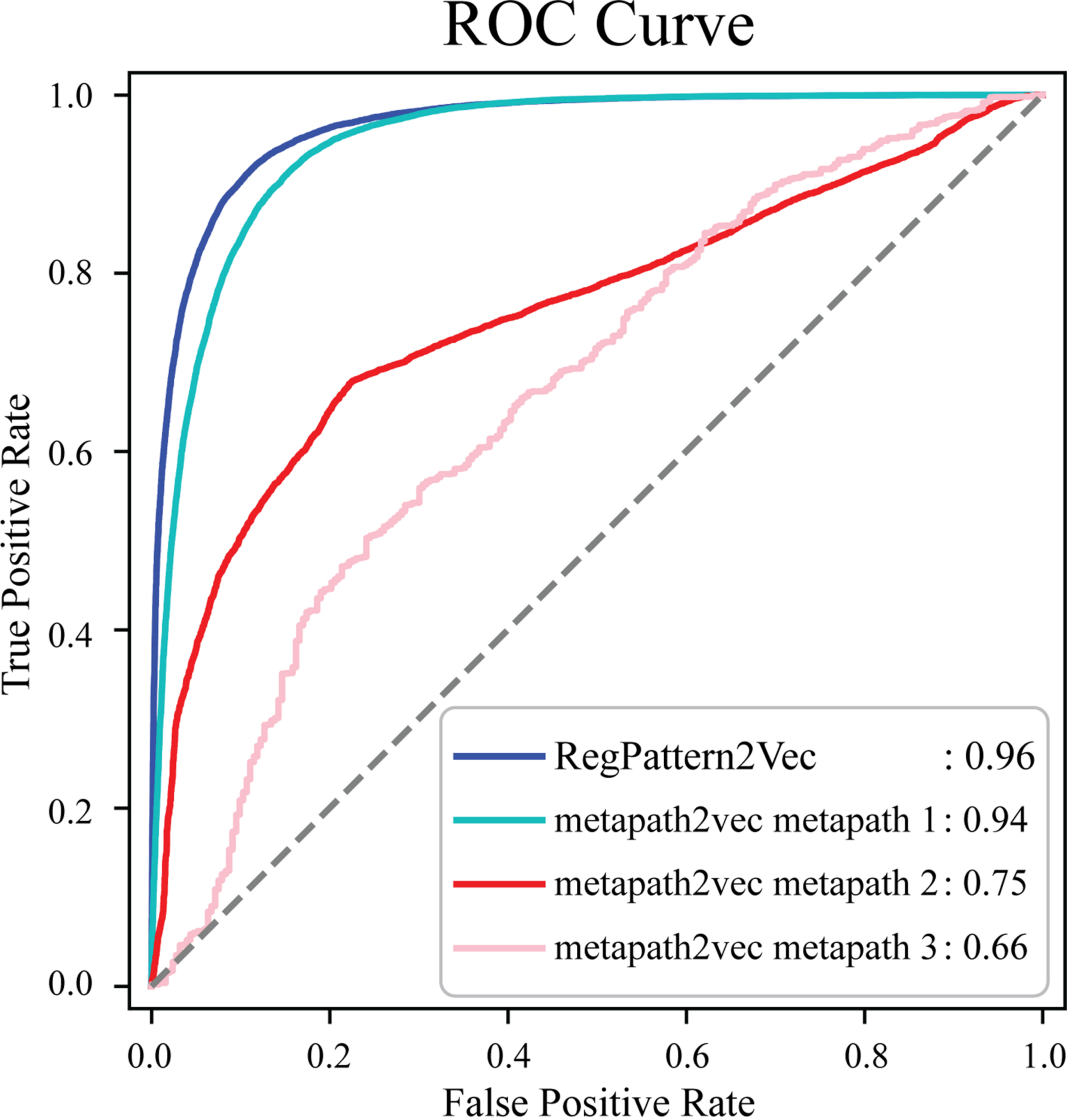
Comparison of AUC ROC curves generated with RegPattern2Vec and metapath2vec. The comparison of AUC ROC curves of RegPattern2Vec and differing schemas utilized with the metapath2vec model. AUC ROC curves were generated by excluding 50% of the known associations from the training set, link prediction was then carried out using a logistic regression algorithm type as the binary classification model. metapath 1 consists of the schema [’*Protein*’, ’*FunctionalDomain*’, ’*Protein*’, ’*Pathway*’, ’*Protein*’]. metapath 2 consists of the schema [’*Protein*’, ’*DarkKinase*’, ’*Protein’*, ’*Pathway*’, ’*Protein*’]. metapath 3 consists of the schema [’*LightKinase*’, ’*Protein*’, ’*DarkKinase*’, ’*Protein*’, ’*Pathway*’, ’*LightKinase*’]. metapath 1 was the best performing schema tested, metapath 2 was still among our top performing schemas tested, and metapath 3 was among the worst performing schemas tested.

To evaluate the accuracy of our method and note any improvements from metapath2vec, a test set was first generated by excluding 50% of the known protein-pathway associations from the training set. The training examples were generated using the process detailed in the Materials & Methods (Link prediction workflow–Link prediction as a classification problem’). To compare our method to metapath2vec, we produced AUC ROC curves for both models. Multiple curves were generated for the metapath2vec model as multiple meta-paths were used resulting in variability in the model’s performance (Fig. 4). Metapath 1 (*‘Protein’*, *‘FunctionalDomain’*, *‘Protein’*, *‘Pathway’*, *‘Protein’*) showed the best performance, based on 10-fold cross-validation, with an f1-score of 0.87 and AUC ROC of 0.94 for protein-pathway prediction. In contrast, RegPattern2Vec achieved an f1-score of 0.90 and AUC ROC of 0.96 on the same training and testing datasets (Fig. 4). We also compared RegPattern2Vec to other recently proposed embedding approaches for heterogenous graphs, namely BoxE (Abboud et al., 2020), MAGNN (Fu et al., 2020), and RHINE (Shi et al., 2020). These models were chosen mostly due to their ability to scale with larger knowledge graphs. Surprisingly, these models displayed poor performance in the task of predicting dark kinase pathway associations (Fig. S1), presumably because of the sparsity of knowledge related to dark kinases in the KG. RegPattern2Vec overcomes some of the challenges imposed by data sparsity by resampling a node as a starting point, allowing for constraint backward walking for a greater sampling of neighbor nodes, and use of inverse node degree as a consideration to select the next node in the walk.

### Investigating the impact of hyperparameters on regpattern2vec’s predictive variability among replicate runs

Although RegPattern2Vec is better suited for the task of learning on sparse biological KGs, due to the nature of the random walks, there is an intrinsic level of variability with the predictions made by our model. Although the exact same sequence of nodes will not be sampled each time, there should be a larger context within the embedding space that identifies patterns useful for predictions for the dark kinases sampled. Therefore, overlaps in the *Pathway-Protein* predictions made by our model using different hyperparameters are expected. To demonstrate our model’s robustness, we measured the predictive consistency while modifying hyperparameters across three replicates (runs that all have the same hyperparameters) and compared these results. The percentage of protein-pathway predictions that overlap (within all replicates) was used as a metric for our model’s robustness. This analysis also revealed the variability in the overlap of the protein-pathway predictions on a per-kinase basis (for both light and dark kinases). This variability may be due to imbalances in the data available for each kinase. Notably, greater variability was seen amongst dark kinases, with the highest percentage overlap being 87.5% and the lowest percentage overlap being 0% (for the hyperparameter 40 NW with 95% confidence cutoff). A supplementary table (Table S2) provides further information indicating the overlap seen for each kinase. Additionally, we provided a supplementary figure (Fig. S2), displaying the confidence (0.6 to 1.0) in overlap for all hyperparameters tested.

One source of variability within our model is directly controlled by the hyperparameter “a *number of walks* (NW)”, which specifies the number of times a node should be sampled as the starting node during the random walk process (refer to Materials & Methods ‘Vectorization of nodes and deep learning of semantic relationships between proteins and pathways’). Repeating the walk from a specified node allows for a more complete characterization of neighbor nodes, often allowing for more node/edge types to be captured and considered in downstream analysis which is especially important when working with sparse data.

**Figure 5:**
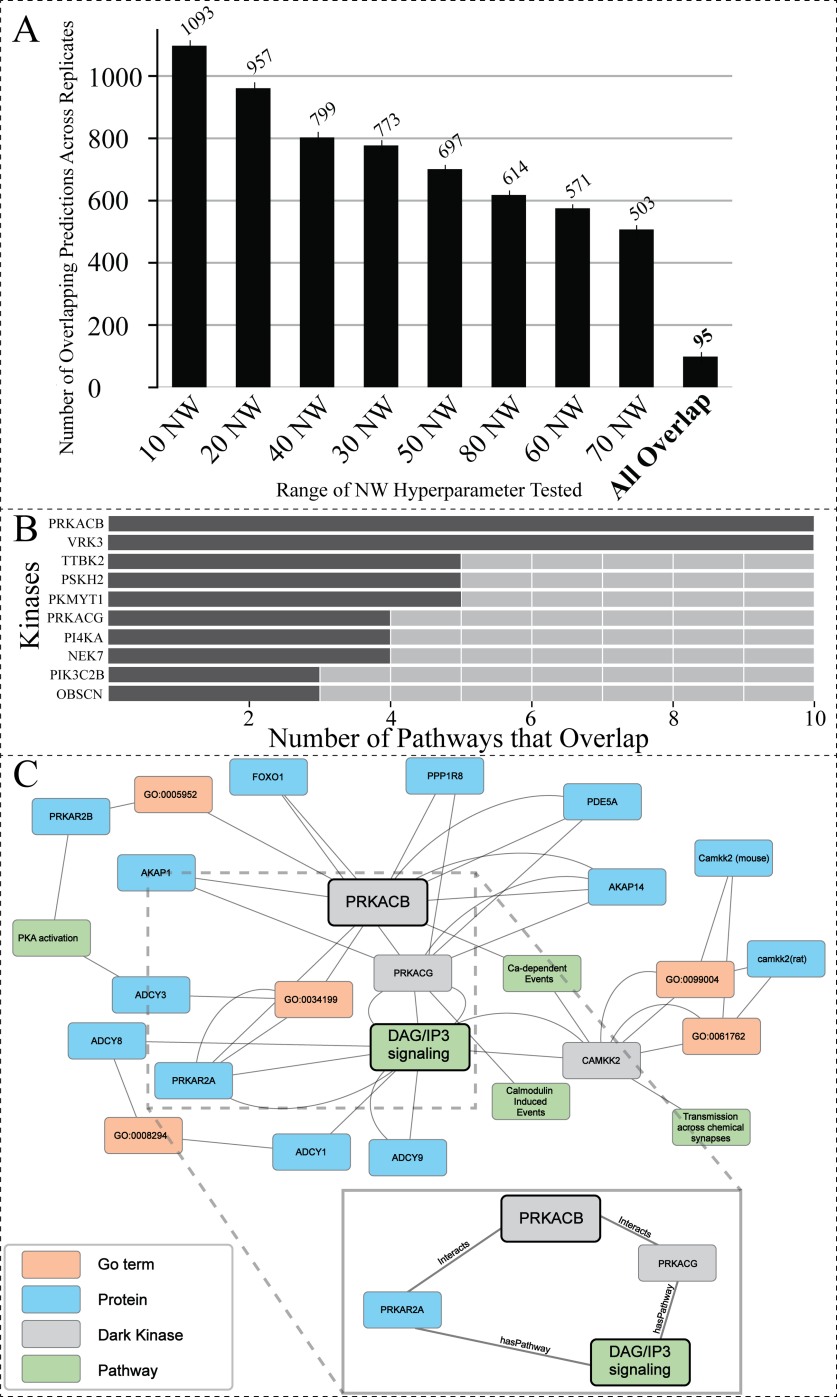
Investigating the overlap among all replicates and NW hyperparameter values. (A) Total number of overlapping *DarkKinase-Pathway* predictions for all variations of the NW hyperparameter tested (10–80) with replicates (each hyperparameter tested three times) for a total of 24 datasets compared. Replicates were averaged and then graphed. Finally, overlap between all 24 datasets was compared in the “All Overlap” bar (B) Graph depicting the number of overlapping pathways for the top ten (out of 34) kinases ranked by number (abundance) of overlapping predictions (C) We highlight a portion of the subgraph for the nodes collected through our modified random walk process for the protein-pathway prediction generated through link prediction between *PRKACB* and *DAG/IP3* signaling. The nodes shown are common between five paths in the KG, obtained through the variation in NW datasets described above. PRKACB was chosen for this analysis based on panel B. The key indicates the color-coded node types, and the magnified panel highlights the highly connected hubs and their edge types.

To find the optimal NW, the same starting node was used for generating paths in the random walk anywhere from 10 to 80 times. This process was repeated, resulting in a total of three replicates for each variation of the NW hyperparameter. Overlap between the protein-pathway predictions obtained through replicate hyperparameter conditions was then compared. From this analysis, we sought to identify the smallest value for the hyperparameter NW that can accurately capture local structure and node context within the graph to make consistent predictions (as determined by replicate overlap). This analysis revealed that changing the hyperparameter NW did not drastically change the number of overlapping protein-pathway predictions between replicate datasets (Fig. S3). Of note, the value of 40 for the hyperparameter NW (abbreviated 40 NW) generated the most consistent predictions among the NW parameters tested, with a mean of ∼22% overlapping pathways across three 0.7 cutoff replicates when averaging overlap over all kinases even when utilizing a range of confidence cutoffs from 0.7−0.95 (Fig. S3). As a result of this benchmark, we determined that 40 NW is the optimal hyperparameter for predicting protein-pathway relationships in the current KG, as it performs slightly better (as measured by replicate overlap) than the more computationally intensive hyperparameter values tested.

### Hyperparameter optimization results in consistent replicate pathway predictions for 34 dark kinases across different replicate runs

After finding the optimal value for the hyperparameter NW (Fig. S3), we decided to investigate the overlap of all protein-pathway predictions produced by our previous analysis in which we test the hyperparameter NW (from 10–80 with a total of three replicate runs for each condition). The overlapping predictions present amongst highly variable walks suggest that these predictions are made based on nodes more often sampled within our network. After filtering for predictions that have over 0.95 confidence, only 95 dark kinase protein-pathway predictions emerged that were present in all replicates of all variations of NW tested (a total of 24 datasets) (Fig. 5A), resulting in representative protein-pathway predictions for 34 of the unique dark kinases in our dataset (Table S3). The top ten kinases (ranked according to the number of predictions that overlap) are shown in Fig. 5B. Of note, many of the protein-pathway predictions generated by our model were highly consistent (either in pathways with similar function or protein family involvement) on a per kinases basis (Table S3). For example, for the dark kinase VRK serine/threonine kinase 3 (VRK3), seven out of ten predictions were related to Toll Like Receptor (TLR) cascades, and five out of five protein-pathway predictions for the dark kinase protein kinase, membrane-associated tyrosine/threonine 1 (PKMYT1) were related to cell cycle control (Table S3). We further focused on a subset of dark kinases for path analysis described below.

### Path analysis provides context for prediction associations between understudied PRKACB and DAG/IP3 signaling

The dark protein kinase cAMP-activated catalytic subunit beta (PRKACB) was amongst the kinases identified with the highest overlap of protein-pathway predictions (Fig. 5B). PRKACB, the beta-catalytic subunit of Protein Kinase A (PKA), appears to be involved in calcium regulation and modification, a known role for PKA (Barodia, Sophronea & Luthra, 2022; Kania et al., 2017; Ould Amer & Hebert-Chatelain, 2018; Palencia-Campos et al., 2020). Eight of the ten pathways identified were directly linked to calcium regulation (Table S3). To contextualize the predictions made by our model for PRKACB, we created a subgraph displaying common nodes traversed during the random walk process for the node representing the Reactome protein-pathway prediction R-HSA-1489509 (*DAG/IP3Signaling*). A portion of the subgraph highlighting the common nodes sampled between different paths are shown in Fig. 5C. The nodes found in all sampled paths are more likely to represent nodes that are important for providing the context that results in the shared protein-pathway prediction. The entirety of the subgraph (Data S1) displays all nodes with the addition of unconnected nodes that only appear in one path generated during the random walk process.

The highly sampled nodes of the subgraph shown in Fig. 5C, reveal that *PRKACG* (protein kinase cAMP-activated catalytic subunit gamma), *PRKAR2A* (protein kinase cAMP-dependent type II regulatory subunit alpha) (Manchev et al., 2014; Wei et al., 2021) and (calcium/calmodulin dependent protein kinase kinase 2) *CAMKK2* (Najar et al., 2021) are nodes often sampled for the *DAG/IP3Signaling* prediction. The nodes being sampled multiple times demonstrate our model’s ability to leverage PPIs to infer pathway involvement. Additional nodes of interest that are often sampled for this prediction include PPIs between PRKACB (protein of interest) and both A-kinase anchor protein 28 (AKA28), and cGMP-specific 3′, 5′-cyclic phosphodiesterase (PDE5A). Both proteins have biologically relevant functions supporting the protein-pathway prediction for PRKACB’s involvement with DAG/IP3 signaling. AKA28 has been shown to bind to type II regulatory subunits of PKA (related to our dark kinase) and anchors/targets PKA to discrete locations within the cell (Kennedy & Scott, 2015; Kultgen et al., 2002; Omar & Scott, 2020; Sarma et al., 2010). PDE5A is a phosphodiesterase that regulates intracellular levels of cAMP and AMP (Peng et al., 2020). These observations support the predicted link/association between understudied PRKCB kinase and DAG/IP3 signaling pathway.

### Predicted association of understudied CDK19 in TGF-beta receptor signaling: interpretability with path analysis and validation with PPI datasets

**Figure 6:**
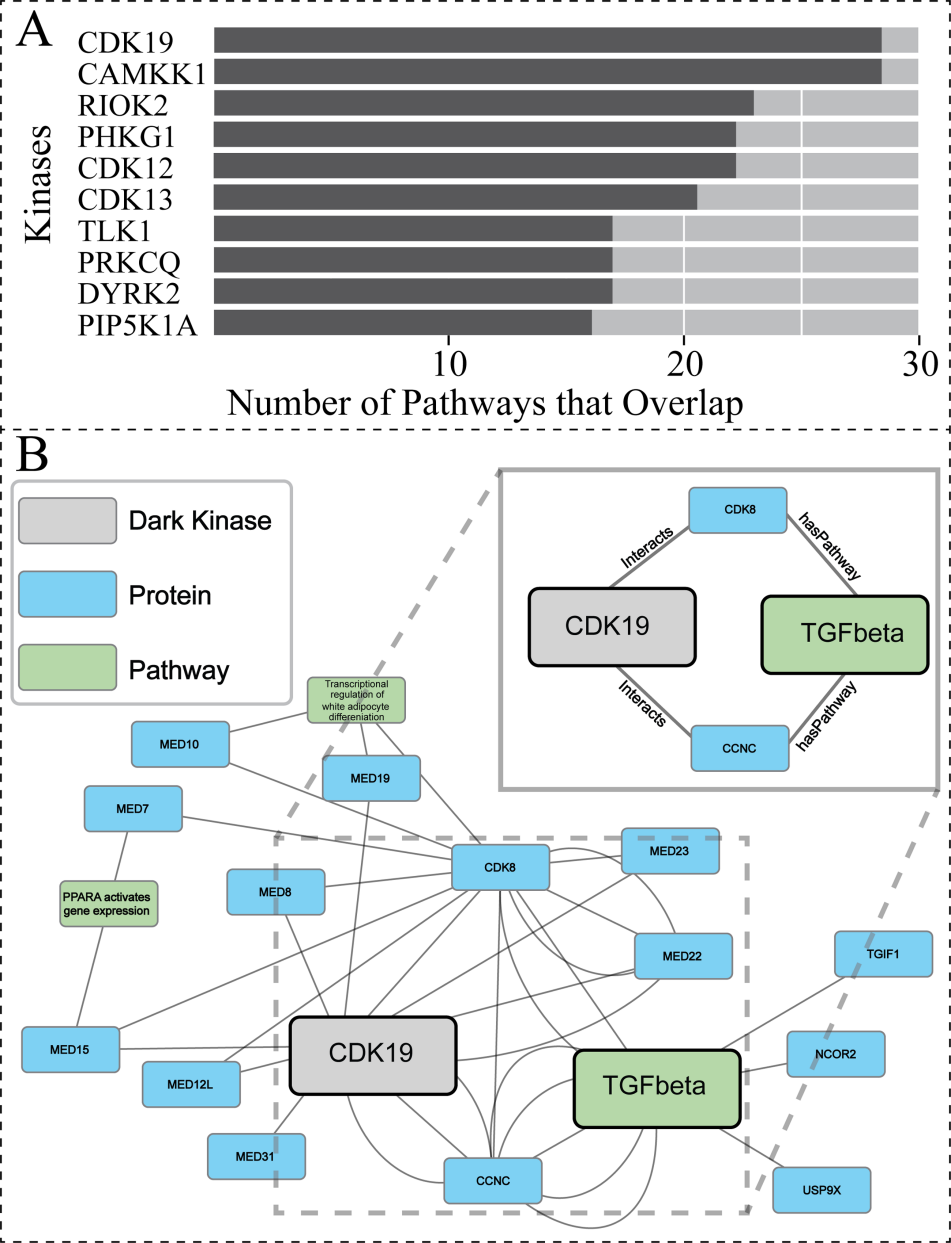
Investigating protein-pathway predictions and dark kinase knowledgebase experimentally validated predictions. Protein Interaction and Proximity (PPI) data for dark kinases obtained through the Dark Kinase Knowledgebase were used as input for Reactome enrichment. These predictions were then compared to those produced by our model using data produced during the hyperparameters exercise (24 datasets). (A) Graph depicting the number of overlapping pathways between the two datasets for the top ten kinases ranked by number (abundance) of overlapping predictions. (B) We highlight a portion of the subgraph for the nodes collected through our modified random walk process for the protein-pathway prediction generated through link prediction between *CDK19* and *TGF-beta*. The nodes shown are common between five paths in the KG, obtained through the 40 NW dataset. CDK19 was chosen for this analysis based on (A). The key indicates the color-coded node types, and the magnified panel highlights the highly connected hubs and their edge types.

To further explore the biological relevance of our predictions for dark kinases, we compared our protein-pathway predictions to Reactome datasets generated using Protein Interaction and Proximity (PPI) data for dark kinases not included in our training data. PPI data was obtained from the Dark Kinase Knowledgebase (Berginski et al., 2021). Of note, many kinases in this dataset did not have sufficient protein-interactors to perform Reactome pathway enrichment analysis. For the 50 dark kinases with an adequate number of identified interactors, the overlap between the protein-pathway predictions generated by our link prediction and the Reactome enrichment generated by PPI data were compared. Figure 6A shows the top ten dark kinases ranked according to protein-pathway prediction overlap abundance and Table S4 provides the pathway information for all dark kinases with overlap seen. Among the dark kinases with the highest overlap from this comparison was cyclin-dependent kinase 19 (CDK19), a dark kinase belonging to the cyclin-dependent kinase superfamily (Malumbres, 2014).

After performing similar subgraph analysis to that previously described for PRKACB (Results,, ‘Hyperparameter optimization results in consistent replicate pathway predictions for 34 dark kinases across different replicate runs’), we were able to isolate nodes commonly traversed for the protein node of interest (*CDK19*) and the pathway node of interest *TGF-betaReceptorSignaling* (R-HSA-170834) (Fig. 6B). Again, only a subset of the paths is shown for ease of viewing. The entirety of the subgraph (Data S2) displays all nodes with the addition of unconnected nodes that only appear in one path generated during the random walk process. For the dark protein kinase CDK19 with the link prediction of TGF-beta receptor signaling, two “key” node hubs were identified including *CCNC* (cyclin-c) and *CDK8* (cyclin dependent kinase 8). Recent total mRNA sequencing has demonstrated that loss of CCNC could activate the transforming growth factor (TGF)-beta signaling pathway (Tang et al., 2021). CDK8 has also been implicated in TGF-beta signaling through previous studies showing they drive Smad transcriptional action and turnover in TGF-beta pathways (Alarcón et al., 2009). Through interactions with several members of the mediator of RNA polymerase II transcription (MED) protein family, CDK19 can also indirectly exploit the relationship between CDK8 and TGF-beta receptor signaling. It appears that CDK19, and CDK8 have been shown to directly interact with not just each other but MED12, as well as CCNC, forming a complex collectively termed the “Mediator Complex” (Fant & Taatjes, 2019). This complex interacts with DNA-bound transcription factors and RNA polymerase II (Pol II) to activate and repress gene expression, with mediator subunit MED12 promoting TGF-beta signaling through both canonical regulation of transcription and non-genomic activity (Weber & Garabedian, 2018). Taken together this suggests that while direct connections contribute to the predictive power of the model the surrounding node context can also reinforce these connections and can contribute to predictions made.

### Predicted association between understudied PSKH2 and cilium assembly

Protein-pathway predictions were additionally produced by our model for an understudied protein kinase PSKH2 (Byrne et al., 2022) which is a paralog of the Golgi-associated protein serine kinase H1 (PSKH1) (Brede et al., 2000). Currently, the Dark Kinase Knowledgebase (Berginski et al., 2021) lists only ten known protein interactors for PSKH2 and no pathway annotation for this kinase exists in the literature. We utilized this and another recently published dataset (Byrne et al., 2022) not included in training, for the Reactome enrichment analysis and independent validation of RegPattern2Vec predictions on PSKH2.

**Figure 7:**
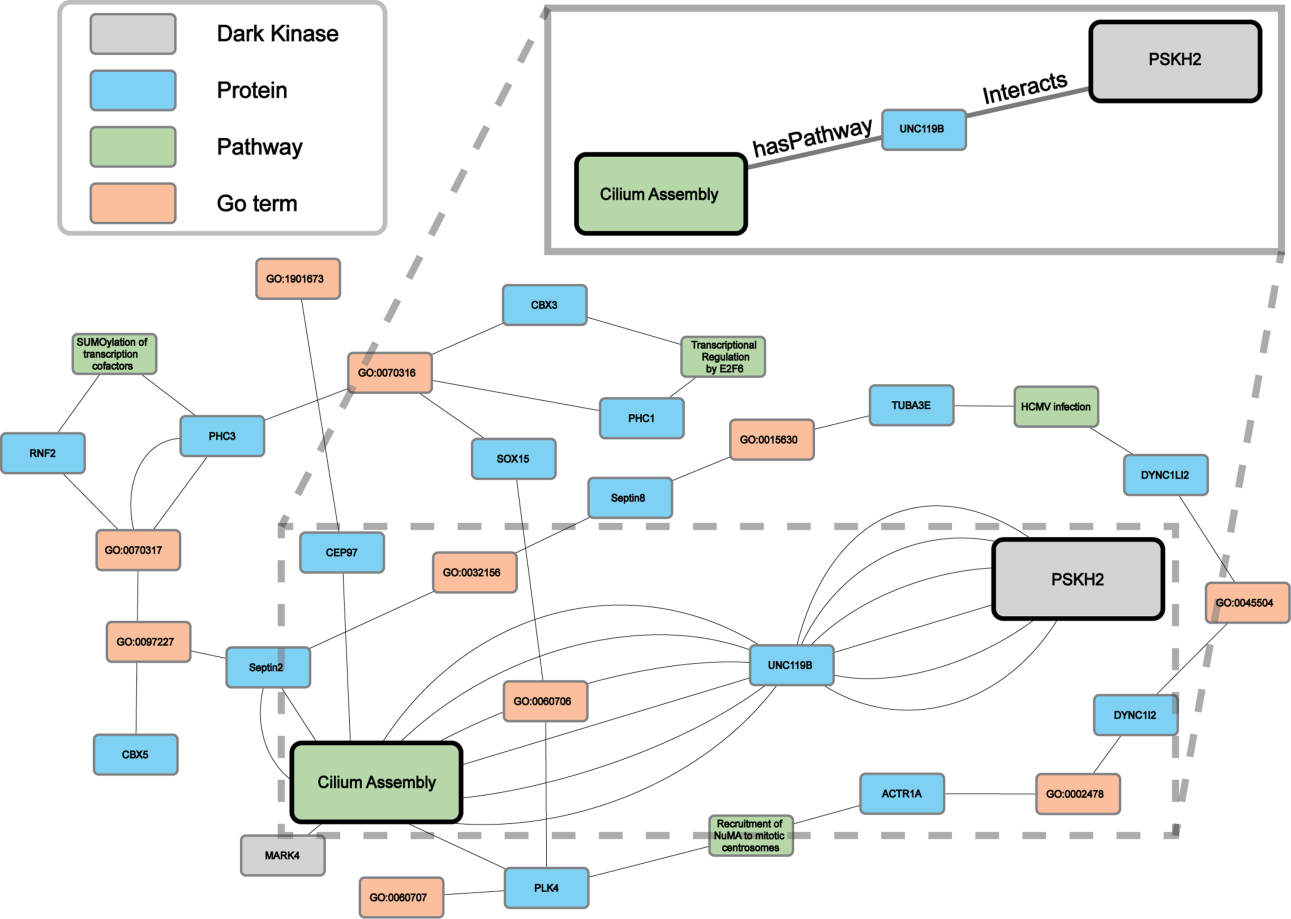
A subgraph of the network context of the KG surrounding PSKH2. As demonstrated in the previous Fig. 5B our model can generate consistent predictions for dark kinase PSKH2. We compared the predictions produced by our model (40 NW) and Reactome enrichment using unpublished mass-spectrometry data produced by our collaborators. We highlight a portion of the subgraph for the nodes collected through our modified random walk process for the protein-pathway prediction generated through link prediction between *PSKH2* and *CiliumAssembly*. The nodes shown are common between five paths in the KG, obtained through the 40 NW dataset. The key indicates the color-coded node types, and the magnified panel highlights the highly connected hubs and their edge types.

Comparing the overlap in protein-pathway predictions (40 NW dataset) with the Reactome enrichment, 18 pathways were shown to overlap (Table S5). The previously characterized subgraph visualization (Results, ‘Hyperparameter optimization results in consistent replicate pathway predictions for 34 dark kinases across different replicate runs’) was then performed, with five random paths chosen for visualization of the nodes commonly traversed for the protein node of interest (*PSKH2*) and the prediction node of interest (*CiliumAssembly* R-HSA- 5617833) (Fig. 7 and Data S3). Through this analysis one major “key” node hub was identified, *UNC119B*. unc-119 lipid binding chaperone B (UNC119B) is a protein that plays a key role in the localization of ciliary proteins by binding to N-myristoylated proteins and masking the hydrophobic lipid from hydrophilic cytosol, therefore facilitating trafficking to the primary cilium or the immunological synapse (Stroukov et al., 2019; Yelland et al., 2021). Many of the additional commonly traversed nodes belong to larger families of proteins known to play important roles in cilium assembly such as tubulins and dyneins. Thus, further characterization of these proteins can shed light on the role of PSKH2 in cilium assembly.

Overall, through our analysis of the paths utilized by the model as context for the link prediction process, we demonstrated that our modified random walk approach, RegPattern2Vec, can successfully sample multiple types of nodes and edges. These findings support our notion that our model preserves both local and latent structure through appropriate neighbor sampling and therefore provides the node context needed to make predictions. The predictions made by our model regarding dark kinases allow us to explore dark kinases of interest in a pathway context, giving us an idea of how these kinases may function and relate to other proteins.

## Discussion

In this work, we present a network approach to predict pathway associations for understudied dark kinases by leveraging the network context of well-studied kinases. We predict pathway associations for dark kinases and propose a new graph embedding approach, RegPattern2Vec, which has significant advantages over the previously proposed metapath2vec approach for KG embedding. First, unlike metapath2vec, RegPattern2Vec does not require pre-defined knowledge of KG schema to define the paths. Second, because RegPattern2Vec does not use fixed length meta-paths, it captures the structure of the graph and the characteristics of both local and latent representations more effectively, as indicated by improved performance measures (Fig. 4). Third, our hyperparameter optimization strategies, combined with analysis of meta-paths, enable biological interpretation of predicted links.

While the KG embedding and ML approaches described here have distinct advantages over metapath2vec for link prediction, our model can be further improved through the generation of curated training and testing datasets and additional data that capture pathway context. Currently, to generate negative datasets, any missing link between nodes is used as an indication that a relationship does not exist. In short, the negative sample is simply non-connected nodes. In certain knowledge domains turned into KGs this is appropriate, however just because a link does not exist within our graph does not mean it does not occur in a cellular context (*i.e.,* two proteins interacting or a protein having a certain association). To address this limitation, we are currently working on a KG that includes subcellular localization information of proteins, and tissue/cell specificity to create a more informed negative set that better reflects the natural boundaries placed by biology rather than the boundaries of our current knowledge. Additional future directions include fully incorporating edge information into the embeddings used for our model’s predictions. The use of edge labels could allow for the use of additional Natural Language Processing (NLP) based models such as Transformers (Bi et al., 2022; Koncel-Kedziorski et al., 2019; Xie et al., 2022; Yao, Mao & Luo, 2019).

Overall, we have demonstrated that our model can make valid biological predictions even with sparse input through the use of node context extracted from the KG. While this current work was focused on placing dark kinases in a pathway context, within the KG are nodes and edge types relevant for PPI and substrate predictions as well (Fig. 1C, Table S1). Therefore, our KG can be further utilized for other predictive tasks and the functionality of the KG can be further expanded by incorporating other forms of data. For example, including information such as cell-type specific expression of kinases could potentially improve the overall model performance while enabling predictions for cell-type specific dark kinase functions and interactions. However, the addition of new data types can significantly alter the topology of the KG. As data is added, new node/edge types and schemas will need to be incorporated into the graph and hyperparameters will again need to be optimized. The protocol described in Results (‘Benchmarking RegPattern2Vec’s predictive performance with metapath2vec and other graph embedding approaches’) for testing hyperparameters serves as a framework for continued usage of the KG, and exploration of variability within the random walk process. The code for RegPattern2Vec has been published on GitHub and these methodologies can be applied to additional KGs for other understudied proteins and protein families.

## Conclusions

We sought to characterize understudied dark kinases utilizing a network-based approach that combines multi-domain knowledge on both well-studied kinases and dark kinases within a single heterogeneous KG. Our method focuses on improvements within the random walk component of the graph embedding process. To accomplish this, we utilized RegPattern2Vec, allowing for the selection of the relevant part(s) of the KG using labeled semantics (regular patterns) to not only learn the local similarity between nodes and edges but also more complex and non-trivial associations which lead to novel protein-pathway predictions for dark kinases. Our method performs better in terms of scalability for large graphs compared to other network-based methods by sampling the graph more accurately while preserving node/edge context and structure.

##  Supplemental Information

10.7717/peerj.15815/supp-1 Supplemental Information 1 Comparing AUC ROC Curves of RegPattern2Vec and differing models for link prediction utilizing embeddings AUC ROC curves were generated by excluding 50% of the known associations from the training set, link prediction was then carried out using a logistic regression algorithm type as the binary classification model. Both RegPattern2Vec and metapath2vec (with the best performing schema) perform well despite the imbalanced nature of the data within the graph which causes both data sparsity and scarcity. BoxE (fully expressive proximity-preserving method), RHINE (method that uses two major meta-labels for preserving graph structure), and MAGNN (a GNN based method that uses one-skip and two-skip metapaths- a global sampling technique and node aggregation) appear to have greater difficulty learning on our knowledge graph. All models were used with default settings, the only parameters changed were embedding size (MAGNN, RHINE. BoxE, metpath2vec) to allow for use of the embedding with our link prediction method. The configuration files which show the parameters each model was run with are additionally included in our github ( https://github.com/gravelCompBio/RegPattern2Vec).

10.7717/peerj.15815/supp-2 Supplemental Information 2 Confidence Values for Replicates with Varying Hyperparameters Confidence ranges from 0.60 to 1.00 for all hyperparemeters tested (NW 10-80 with each hyperparameter tested three times) are displayed. The *y*-axis depicts the number of overlapping predictions averaged between replicates while the *x*-axis depicts and each hyperparameter number of walk is displayed as a separate graph.

10.7717/peerj.15815/supp-3 Supplemental Information 3 Measuring Variability Among Hyperparameter Replicates Comparison of the overlap between protein-pathway predictions produced by the model when changing the hyperparameter for Number of Walks (NWs). The NW hyperparameter, indicating how many times the starting node should be sampled, was tested from 10 to 80 with each variation of the changed NW hyperparameter for the random walk process replicated three times. (A–B) Box plot of the average percentage overlap for protein-pathway predictions between the three replicates produced for all variations of the hyperparameter used (10–80). Overlap of the predictions produced by the model was compared on a per dark kinase basis and then averaged overall for all dark kinases (A) filtered for 0.7 confidence or (B) filtered for 0.9 confidence.

10.7717/peerj.15815/supp-4 Supplemental Information 4 Node Statistics Table depicting all node type abundance and average degree (number of edges) for node types within our KG.

10.7717/peerj.15815/supp-5 Supplemental Information 5 Protein-Pathway Predictions Overlap for all Dark Kinases Table depicting the average overlap between three replicates for all hyper-parameters tested (NW from 10–80). Columns include “Number of predictions that overlap across 3 replicates” and “Total number of unique pathway predictions”. The “Percent of pathway predictions that overlap” column is made by dividing the values of the other columns.

10.7717/peerj.15815/supp-6 Supplemental Information 6 Protein-Pathway Predictions that Overlap with All NW Replicates This file can be used to generate the subgraph highlighted in Fig. 6 through use with Cytoscape program. Columns are labeled according to their input in Cytoscape. The file includes more nodes that were displayed in the figure. More information on how this data was generated can be found in manuscript (Results ‘Predicted association of understudied CDK19 in TGF-beta receptor signaling: interpretability with path analysis and validation with PPI datasets)

10.7717/peerj.15815/supp-7 Supplemental Information 7 Protein-pathway predictions that overlap with protein–protein interactions provided by the dark kinase knowledgebase Table depicting all protein-pathway predictions produced through our model that overlapped with the Reactome enrichment results produced through protein–protein interactions from the Dark Kinase Knowledgebase. Pathway associations for dark kinases.

10.7717/peerj.15815/supp-8 Supplemental Information 8 Protein-pathway predictions of PSKH2 that overlap with protein–protein interactions generated using mass spectrometry Table depicting all protein pathway predictions that overlapped with the protein–protein interactions from experimentally validated mass-spectrometry data not used in training. Pathway associations that were connected to dark kinases from RegPattern2Vec’s predictions were linked to their associated pathway according to Reactome.

10.7717/peerj.15815/supp-9 Supplemental Information 9 Subgraph of PRKACB generated from random walks sampled that include PRKACB This file can be used to generate the subgraph highlighted in Fig. 5 through use with Cytoscape program. Columns are labeled according to their input in Cytoscape. The file includes more nodes that were displayed in the figure. More information on how this data was generated can be found in manuscript (Results ‘Path analysis provides context for prediction associations between understudied PRKACB and DAG/IP3 signaling’).

10.7717/peerj.15815/supp-10 Supplemental Information 10 Subgraph of CDK19 Generated from Random Walks Sampled that Include CDK19 This file can be used to generate the subgraph highlighted in Fig. 6 through use with Cytoscape program. Columns are labeled according to their input in Cytoscape. The file includes more nodes that were displayed in the figure. More information on how this data was generated can be found in manuscript (Results ‘Predicted association of understudied CDK19 in TGF-beta receptor signaling: interpretability with path analysis and validation with PPI datasets’).

10.7717/peerj.15815/supp-11 Supplemental Information 11 Subgraph of PSKH2 generated from random walks sampled that include PSKH2 This file can be used to generate the subgraph highlighted in Fig. 7 through use Cytoscape program. Columns are labeled according to their input in Cytoscape. The file includes more nodes that were displayed in the figure. More information on how this data was generated can be found in the manuscript (Results ‘Predicted association between understudied PSKH2 and cilium assembly’).

## Additional Information and Declarations

### Data Availability

The following information was supplied regarding data availability: The Knowledge Graph, Example Embedding, UniProt IDs to Gene Names File, Reactome IDs to UniProt ID Mapping File, and Folder Hierarchy Need for Example Code are available at Zenodo: Nathan Gravel. (2023). Supplemental Data 4 (v1.0) [Data set]. Zenodo. https://doi.org/10.5281/zenodo.7541827. The data related to the code are available at Github and Zenodo:
https://github.com/gravelCompBio/RegPattern2Vec
gravelCompBio. (2023). gravelCompBio/RegPattern2Vec: v1.0 (v1.0-alpha.0). Zenodo. https://doi.org/10.5281/zenodo.7541894.
