# Supplementary figures and images for: Predicting protein and pathway associations for understudied dark kinases using pattern-constrained knowledge graph embedding
Source: PeerJ. 2023 Oct 18;11:e15815. doi: 10.7717/peerj.15815 (PMC10590106; doi:10.7717/peerj.15815)

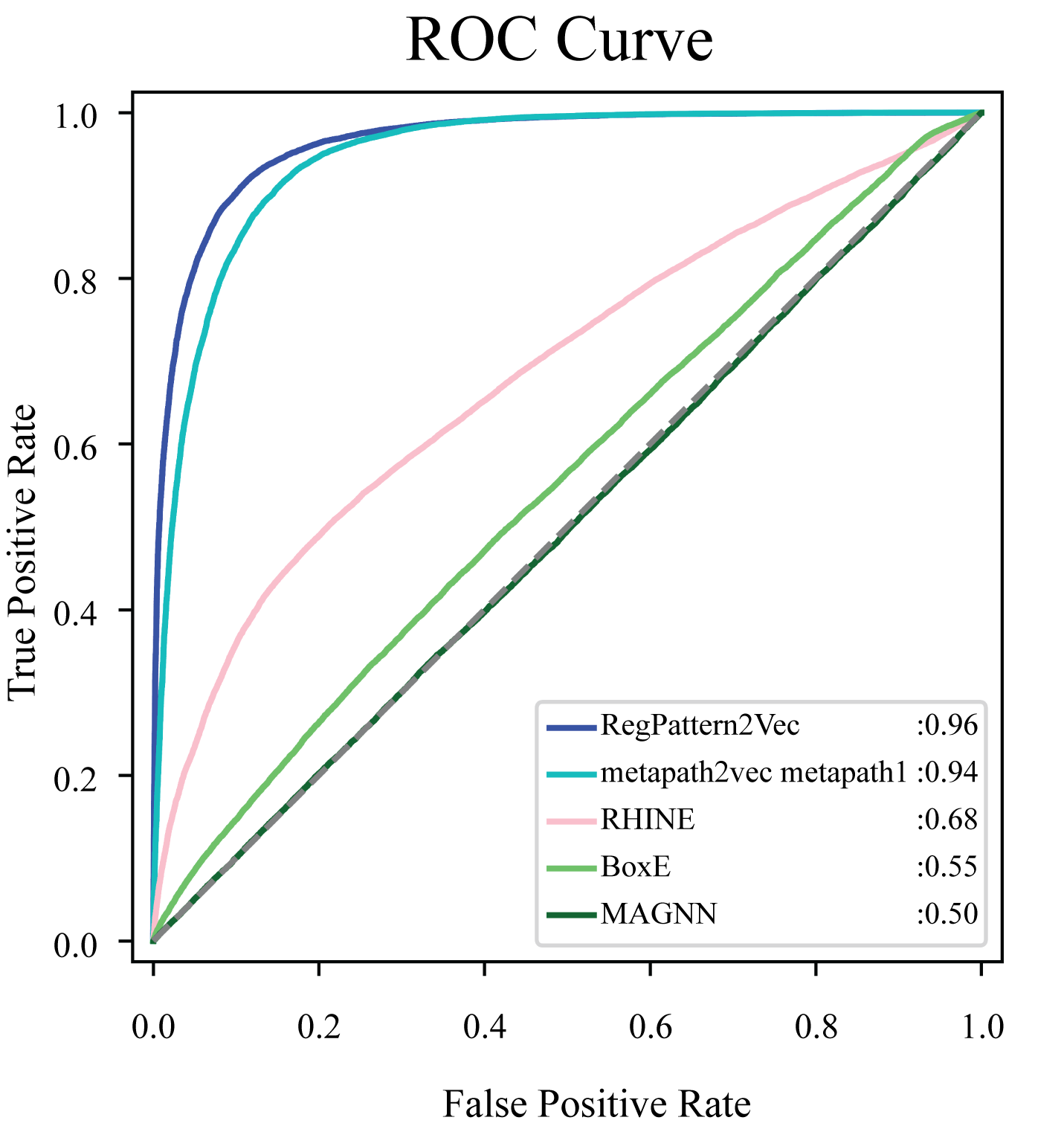

Supplement: Supplemental Information 1 — AUC ROC curves were generated by excluding 50% of the known associations from the training set, link prediction was then carried out using a logistic regression algorithm type as the binary classification model. Both RegPattern2Vec and metapath2vec (with the best performing schema) perform well despite the imbalanced nature of the data within the graph which causes both data sparsity and scarcity. BoxE (fully expressive proximity-preserving method), RHINE (method that uses two major meta-labels for preserving graph structure), and MAGNN (a GNN based method that uses one-skip and two-skip metapaths- a global sampling technique and node aggregation) appear to have greater difficulty learning on our knowledge graph. All models were used with default settings, the only parameters changed were embedding size (MAGNN, RHINE. BoxE, metpath2vec) to allow for use of the embedding with our link prediction method. The configuration files which show the parameters each model was run with are additionally included in our github ( https://github.com/gravelCompBio/RegPattern2Vec). [file peerj-11-15815-s001.png]

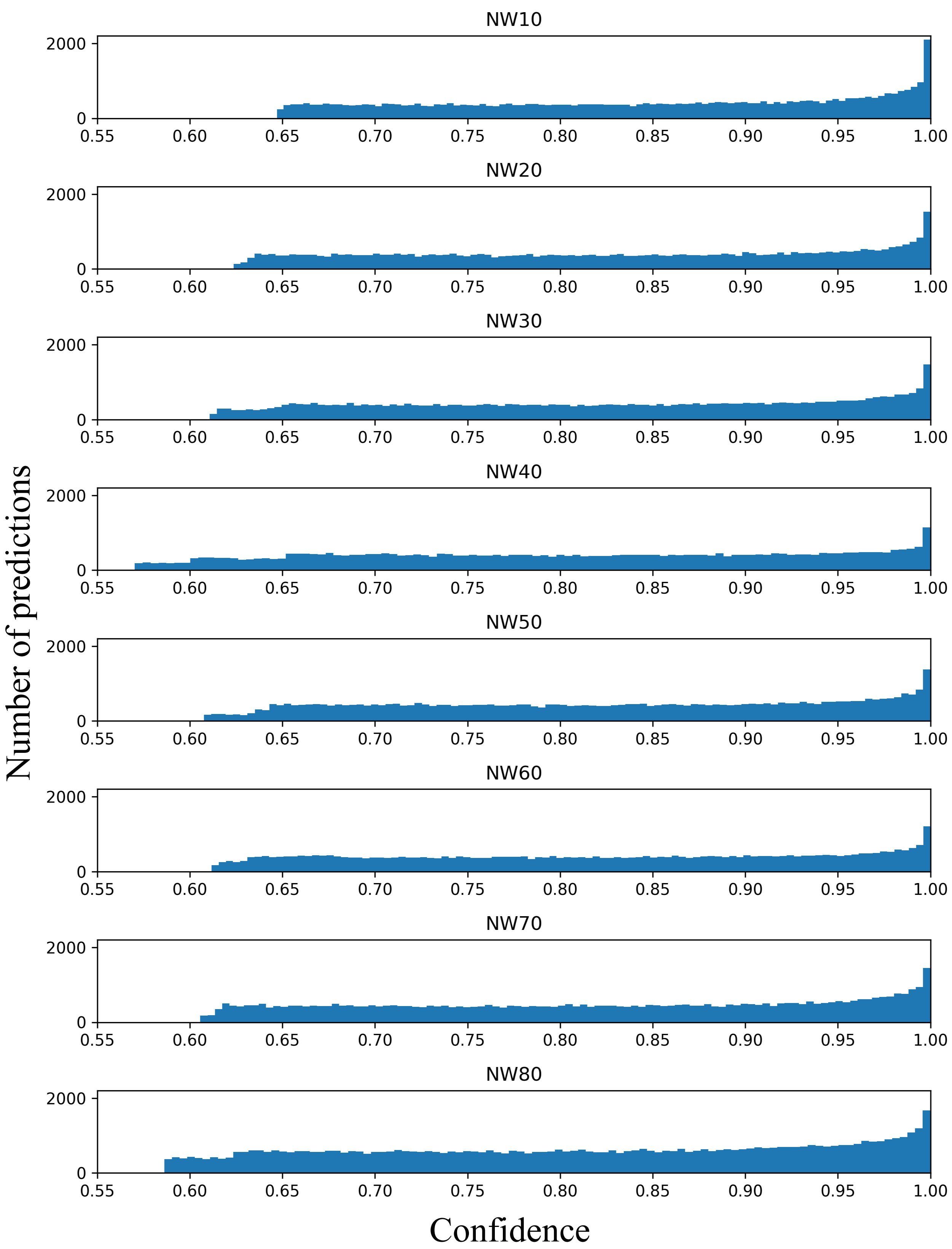

Supplement: Supplemental Information 2 — Confidence ranges from 0.60 to 1.00 for all hyperparemeters tested (NW 10-80 with each hyperparameter tested three times) are displayed. The y-axis depicts the number of overlapping predictions averaged between replicates while the x-axis depicts and each hyperparameter number of walk is displayed as a separate graph. [file peerj-11-15815-s002.png]

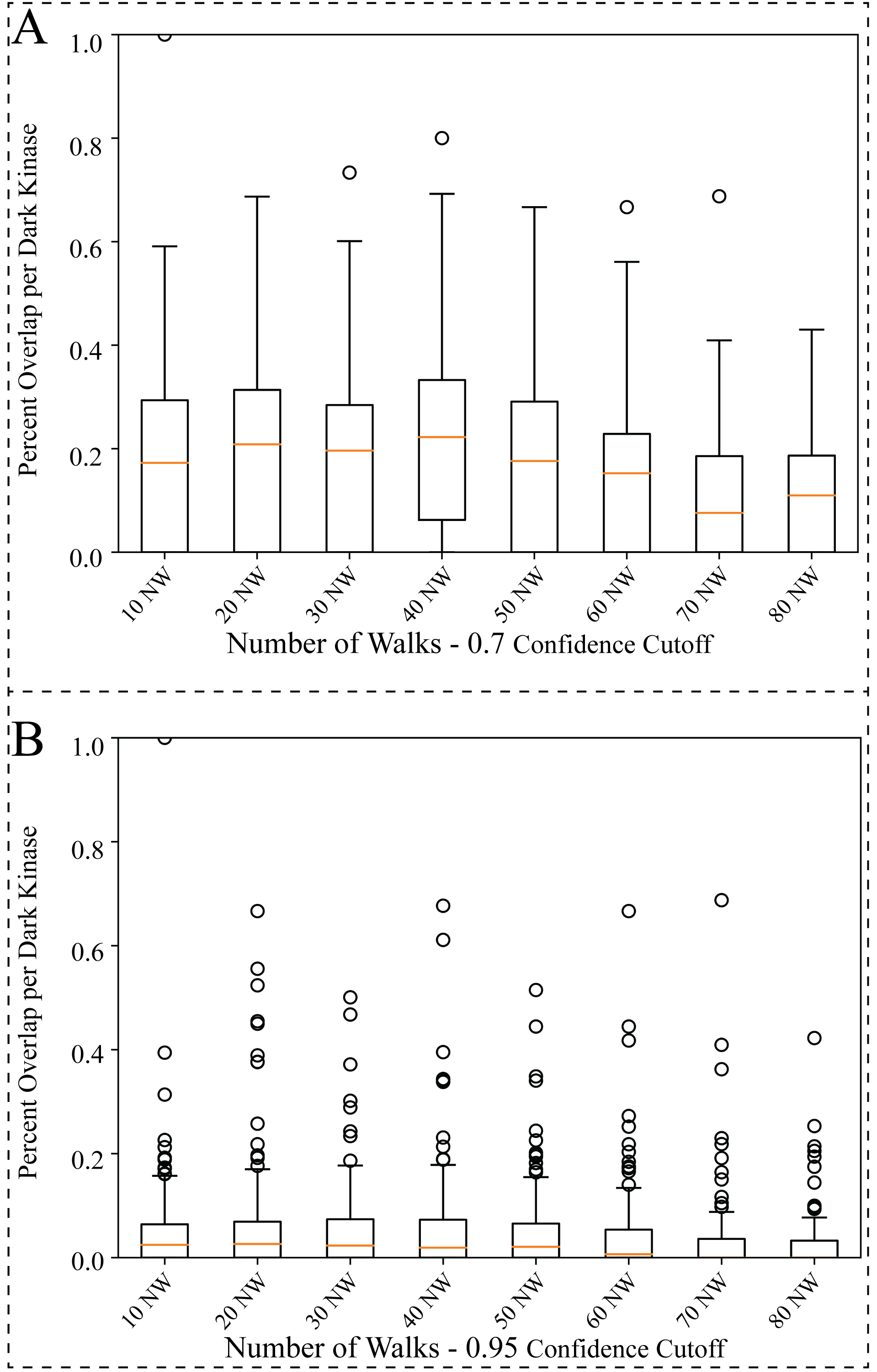

Supplement: Supplemental Information 3 — Comparison of the overlap between protein-pathway predictions produced by the model when changing the hyperparameter for Number of Walks (NWs). The NW hyperparameter, indicating how many times the starting node should be sampled, was tested from 10 to 80 with each variation of the changed NW hyperparameter for the random walk process replicated three times. (A–B) Box plot of the average percentage overlap for protein-pathway predictions between the three replicates produced for all variations of the hyperparameter used (10–80). Overlap of the predictions produced by the model was compared on a per dark kinase basis and then averaged overall for all dark kinases (A) filtered for 0.7 confidence or (B) filtered for 0.9 confidence. [file peerj-11-15815-s003.png]
